# Prelacteal feeding practice and maintenance of exclusive breast feeding in Bihar, India – identifying key demographic sections for childhood nutrition interventions: a cross-sectional study

**DOI:** 10.12688/gatesopenres.12862.3

**Published:** 2019-06-14

**Authors:** Aritra Das, Guntur Sai Mala, Ram Shankar Singh, Amlan Majumdar, Rahul Chatterjee, Indrajit Chaudhuri, Tanmay Mahapatra

**Affiliations:** 1 Concurrent Monitoring, Learning & Evaluation, CARE India Solutions for Sustainable Development, Patna, Bihar, 800013, India; 2 Project Concern International, Patna, Bihar, 800001, India

**Keywords:** Prelacteal feeding, Exclusive breastfeeding, India

## Abstract

**Background**:  Exclusive breastfeeding (EBF) during the first six months of life is considered a high impact, but low-cost, measure for improving nutritional status, and reducing morbidity and mortality among children. However, providing prelacteal feed to a newborn, a widely practiced custom in rural India, is a major barrier to the practice of EBF.  The present study evaluated the association between provision of prelacteal feeding and continuation of EBF among children up to 3 months age in Bihar, a resource-poor Indian state.

**Methods**: Data from four rounds of a population-based multi-stage sampling survey, conducted in 8 districts of Bihar between 2012 and 2013, were used for the present analysis. Using simple and adjusted logistic regression modelling, we tested the association of providing prelacteal feeding with two outcome measures - 1) giving only breastmilk during the last 24 hours, and 2) exclusively breastfed (EBF) since birth (excluding the first 3 days of life).

**Results**: Among 10,262 children for whom prelacteal feeding data was available, 26% received prelacteal feeding. About 55% mothers reported that their children were exclusively breastfed, whereas 82% mothers provided only breastmilk to their children during the previous 24 hours. Children who received prelacteal feeding had approximately 60% lesser odds of being breastfed exclusively during the previous 24 hours [AOR = 0.39(0.33-0.47)] and 80% lesser odds of receiving continued EBF since birth [AOR = 0.20(0.17-0.24)].

**Conclusions**: Frontline workers (FLW) provide nutritional counselling to mothers and children of rural India. In order to improve uptake of EBF, the families practicing prelacteal feeding should be identified early and educated on the harmful effects of prelacteal feeding for EBF and subsequently on infant health. Midwives/nurses at the public and private facilities as well as the home birth attendants should also be made aware about the negative effects of prelacteal feed.

## Introduction

Breastfeeding, besides being natural and inexpensive, serves as the ideal source of infant nutrition. It is not only easily digestible and meets the dietary requirements of the newborn but also provides a number of unique biological and psychological benefits to the mother and child ^1^. If an infant is provided only breast milk and no additional food, water, or other liquids (with the exception of medicines, if needed) up to the sixth month of life, then that infant is considered to be exclusively breastfed (EBF) ^2^. A plethora of evidence endorse early initiation and maintenance of EBF till the recommended age as a key intervention against childhood malnutrition, especially for the low- and middle-income countries ^3, 4^. It has been estimated that, globally, optimal breastfeeding and complementary feeding practices have the potential to prevent more than 200,000 infant deaths annually ^5^. However, despite substantial efforts, only about one-fourth of infants worldwide receive EBF for the recommended duration i.e. six months ^6^.

Providing prelacteal feeding, defined as giving something other than breast milk to an infant during the first three days of life, is an established practice in rural India and other developing countries ^7, 8^. As the definition suggests, provision of prelacteal feeding entails that an infant is not exclusively breastfed. Additionally, prelacteal feeding is associated with various other sub-optimal breastfeeding practices such as not giving colostrum to the neonate and delayed initiation of breast feeding ^7^. Therefore, prelacteal feeding is widely recognized as an important determinant of childhood malnutrition and, subsequently, childhood morbidity and mortality ^8^.

Although the uptake of EBF in India has increased during the recent years, it is still far from optimal ^9^. It is often seen that children are put on EBF during initial months of infancy but EBF is not continued till sixth month ^9^. Therefore, understanding the determinants of continuation of EBF is important for identifying the areas of intervention for childhood nutrition programs in India. The present study aimed to determine the association between provision of prelacteal feeding and continuation of EBF among 3 months old infants in Bihar, an impoverished Indian state.

## Methods

CARE India, a non-government organization, in collaboration with the State Government of Bihar, initiated a project named Integrated Family Health Initiative (IFHI) in 2011 with the primary objective of reducing mortality and malnutrition among infants and mothers in Bihar. As part of the evaluation of IFHI, multiple population based cross-sectional surveys were undertaken to ascertain various health and developmental indicators in the state ^10^. In total, five rounds of these surveys (Rounds I-V), using lot quality assurance sampling (LQAS) technique (a small sample survey design based on binomial distribution) ^11^, were conducted in eight districts (from total 38) of Bihar between 2011 and 2013. A two-stage sampling strategy was adopted in each of the survey rounds: 1) from the list of Anganwadi Centers (village-level ‘last mile’ health service delivery points) in each of the 137 study blocks (sub-districts), 19 Anganwadi Centers were selected using probability proportional to size (PPS) sampling; 2) In the next stage, at the selected Anganwadi Center catchment areas, four eligible households were identified through systematic sampling. An eligible household was defined as that containing mothers of infants of four different age strata: 0–2, 3–5, 6–8, and 9–11 completed months (i.e. a child from any of the four age groups had to be present in the surveyed household). The sampling methodology has been described in a previous article ^12^. In the current analysis, we used the information about infants aged 0–2 completed months during Round-II to Round-V of the LQAS survey (Extended data ^13^).

Two separate outcome indicators for EBF were used – 1) exclusive breastfeeding in the last 24 hours (previous day’s morning to current day’s morning), and 2) practice of EBF since birth (excluding the first 3 days of life). We tested the association of providing prelacteal feeding with the two outcome indicators using separate bivariate and multiple logistic regression models. The multiple logistic regression models were adjusted for the following covariates – child’s gender, number of living siblings, caste, religion, economic status of the household, maternal education level and season. Caste-wise, the families were classified into marginalized caste [scheduled castes (SC) / scheduled tribes (ST) / other backward castes (OBC)] and other/general caste. Religion categories were Hindu and non-Hindu. According to the level of education, mothers were classified into three categories – no formal education, school education up to eighth standard, and school education above eighth grade (middle school). Economic status was assessed using an asset index (AI) based on possession of 25 different household items. For calculation of AI, a relative weight was assigned to each of these items and an aggregated score was generated by adding the weighted score for each item possessed by a household. The cumulative asset scores were then log-transformed to create the AI. Based on the percentile distribution of AI, we then created AI tertiles and classified the families according to the AI tertile they belonged to – low, middle and high wealth ^12^. As seasonal variations have been reported to influence breastfeeding practices in rural Bihar ^12^, we further adjusted for the season of data collection. Based on the prevailing weather pattern in Bihar, we classified the interviews conducted during November to February as those conducted in ‘winter’ season, April to August as ‘summer’ and rest of the months as ‘autumn/spring’. All analyses were carried out using the survey data analysis procedures in SAS (version 9.4) using relevant sample weights and incorporating information about multi-stage sampling.

## Results

The current analysis utilized the information on 10,392 infants aged up to 3 months for whom complete information on the relevant parameters was available. The participating households were predominantly Hindu (86%) and about one-fourth (27%) of them belonged to marginalized castes (Scheduled Castes and Scheduled Tribes). Only about 17% families lived in a ‘Pucca’ or brick-built house. Almost two-thirds of the mothers (64%) did not receive any formal education. Characteristics of the surveyed population have been described in detail in a separate publication ^12^. Among these children, 8533 (82.11%) received only breastmilk during the previous 24 hours, while 5713 (54.97%) had been given nothing but breastmilk (excluding ORS and medicines) since the third day after birth. Out of 10,262 children for whom prelacteal feeding data was available, 2686 (26.17%) received some food other than breast milk during the first three days of life. Logistic regression analysis revealed that, compared to those without prelacteal feeding, infants who received prelacteal feeding had approximately 60% lesser odds of being breastfed exclusively during the previous 24 hours (adjusted odds ratio (AOR) = 0.39; 95% confidence interval (CI) = 0.33-0.47) and 80% lesser odds of receiving continued EBF since birth (AOR = 0.20; 95% CI = 0.17-0.24) [ Table 1].

**Table 1.** The association (Odds ratios and 95% confidence intervals) between different predictors and continuation of exclusive breastfeeding among up to 3 months old children. LQAS rounds 2 to 5. Bihar, India. 2012–2014 (n=10,392) *

Predictors			Outcome [Odds ratios (95% CI)]			
		Reference	Breastfeeding exclusively during past 24 hour period		Practice of EBF till date of interview (excluding initial 3 days)	
			Unadjusted	Adjusted **	Unadjusted	Adjusted **
Prelacteal feed given		Not given	**0.37(0.31, 0.44)**	**0.39(0.33, 0.47)**	**0.19(0.16, 0.22)**	**0.20(0.17, 0.24)**
Hindu		Non-Hindu	**1.33(1.06-1.66)**	1.14(0.89-1.45)	**1.21(1.02-1.44)**	1.02(0.84-1.24)
Marginalized		Non-marginalized	**1.49(1.17-1.89)**	1.26(0.97-1.64)	**0.82(0.68-0.99)**	0.87(0.71-1.08)
Mother's education						
	Educated upto standard VIII	Illiterate	**0.79(0.65-0.95)**	0.88(0.72-1.08)	**1.22(1.06-1.41)**	1.12(0.96-1.3)
	Educated above standard VIII		**0.71(0.58-0.87)**	0.8(0.64-1.01)	**1.18(1.01-1.38)**	1.12(0.93-1.35)
Wealth index						
	Highest tertile	Lowest tertile	**0.67(0.55-0.8)**	**0.71(0.58-0.87)**	1.13(0.99-1.29)	1.08(0.92-1.26)
	Middle tertile		0.89(0.73-1.09)	0.89(0.73-1.1)	1.13(0.98-1.29)	**1.15(0.99-1.33)**
Gender of the child		Female	0.92(0.79-1.07)	0.92(0.79-1.08)	**1.18(1.05-1.32)**	**1.21(1.07-1.37)**
Number of living siblings *			1.03(0.97-1.08)	0.99(0.94-1.04)	**0.96(0.92-0.99)**	0.98(0.94-1.02)

*Treated as continuous variable. The odds ratio depicts the change in the estimate with every unit increase in the number of siblings.

**Each predictor was simultaneously adjusted for rest of the predictors. The adjusted models were further adjusted for the season during which interview was conducted

Numbers in bold indicate statistically significant association ( *P*<0.05)

## Discussion

EBF for the first six months has been recognized as a key intervention to meet India’s Millennium Development Goals (MDG) target regarding child malnutrition (MDG-1) and mortality (MDG-4) ^12^. Despite several programmatic measures, rate of increase in the uptake of EBF in India has been slow ^14^. As India moves from the MDGs to the era of more demanding Sustainable Development Goals (SDG), identifying key intervention areas for improvement in EBF is an essential requirement for achieving the targets pertaining to childhood morbidity and mortality.

We found that about a quarter of families in rural Bihar provided prelacteal feed to neonates and those practicing prelacteal feeding were less likely to maintain EBF. Therefore, on one hand, awareness campaigns and other measures against the unwholesome practice of prelacteal feeding need to be reinforced; but more importantly, our findings suggest that the families providing prelacteal feed to neonates constitute a key group for targeted early interventions on EBF. In rural India, a team of ground level health workers called frontline workers (FLW) - comprising of Anganwadi workers (AWW) and Accredited Social Health Activist (ASHA) – help in reaching various services offered under Integrated Child Development Services (ICDS) scheme and National Health Mission programs to the mothers and children. The Auxilliary Nurse Midwives or ANMs are the key health functionary at the Health Sub-centre (HSC) level (consisting of several villages) with a broad set of responsibilities, including the support, local supervision and capacity building of the ASHA and AWW working in respective HSC catchment areas. As these cadre of health workers provide counselling on childhood nutrition e.g. EBF and complementary feeding during their pre- and post-natal home visits, they can be further equipped to intensify their focus on the families that report practicing prelacteal feeding. We recommend efforts to ensure active identification of these families during FLW home visits and to ascertain that they are subjected to EBF counselling and other programmatic measures on EBF maintenance. Another important intervention would be to intensify health education of would-be mothers, especially during pregnancy, and other family members such as their mothers-in-law about the harmful effects of prelacteal feeding for EBF and subsequently on infant health. Targeted education on the same topic for midwives/nurses working at the public and private hospitals and birth attendants conducting home deliveries may also prove useful in reducing the practice of prelacteal feeding.

*Limitations* - There were few limitations in the current study. First, owing to cross-sectional nature of the data we were often unsure about the temporal relation between the study parameters. This limited our ability to draw causal inferences from observed associations between dependent and predictor variables. Second, the information on breastfeeding practices was based on mothers’ report and not actual observation. Thus, there was possibility of social desirability bias as the mothers who were aware about EBF might have reported that they practiced the same even if they did not. The reported nature of data also made our analyses susceptible to recall bias - especially for the data on EBF for full six months. Further, as the mothers of under three month old children were interviewed the ability to recall Prelacteal feeding could vary between mothers of neonates and that of more than two month old children. However, the ability to recall is likely to be non-differential i.e. the recall is unlikely to differ between the mothers practicing EBF and those who were not. Finally, because the study was conducted in an economically deprived region, the findings may not be generalizable to pan-India level and also among families belonging to higher socioeconomic strata.

## Ethics approval

The current study was approved by the `Institutional Committee for Ethics and Review of Research’ of Indian Institute of Health Management Research ( www.iihmr.org), Jaipur, India.

## Consent

Verbal informed consent was obtained from each agreeing participant before the interview and measurements, after explaining the details of the study in a language that they could understand. Given that approximately 60% of the study participants did not have any formal education, the investigators opted for verbal consent instead of written consent.

## Data availability

The data underlying this study and data codebook is available from Open Science Framework.

OSF: Dataset 1. Data for Exclusive breastfeeding - LQAS R2-R5. https://doi.org/10.17605/OSF.IO/FM925 ^13^

This dataset is available under a CC0 1.0 Universal License.

## Extended data

Questionnaires used as part of this study are available from Open Science Framework.

OSF: Extended data. Data for Exclusive breastfeeding - LQAS R2-R5.

File - CARE LQAS Qre 0-2 R5_SA.pdf

https://doi.org/10.17605/OSF.IO/FM925 ^13^

Available under a CC0 1.0 Universal License
