# Supplementary material for: Prelacteal feeding practice and maintenance of exclusive breast feeding in Bihar, India – identifying key demographic sections for childhood nutrition interventions: a cross-sectional study
Source: Gates Open Res. 2019 Jun 14;3:1. Originally published 2019 Jan 8. [Version 3] doi: 10.12688/gatesopenres.12862.3 (PMC6584739; doi:10.12688/gatesopenres.12862.3)
Supplement: Peer review history

# Peer review: Reviewer response for version 3

**DOI:** 10.21956/gatesopenres.14153.r27389

**Published:** 2019-06-17

**Authors:** Anna Lartey

**Affiliations:** 1 Nutrition Division, Food and Agriculture Organization of the United Nations (FAO), Rome, Italy

No further comments.

I have read this submission. I believe that I have an appropriate level of expertise to confirm that it is of an acceptable scientific standard.

# Peer review: Reviewer response for version 2

**DOI:** 10.21956/gatesopenres.14009.r27162

**Published:** 2019-06-04

**Authors:** Anna Lartey

**Affiliations:** 1 Nutrition Division, Food and Agriculture Organization of the United Nations (FAO), Rome, Italy

This is an interesting analysis looking at the practice of prelacteal feeding in relation to EBF practice. Please find below the following edits:

 Abstract:

- Under Methods line 3: Should be “Data … were” not “data was”. (Plural for data.)

Conclusion:

- The study could couch a better conclusion than what is there. The recommendation that “the families practicing prelacteal feeding should be identified early and subjected to intensive counselling by FLWs” is not a practical recommendation and does not address the problem. A more reasonable concluding recommendation would be to intensify education to all would-be mothers, especially during pregnancy and to grandmothers about the harmful effects of prelacteal feeding for EBF and subsequently on infant health. Places of child birth - hospitals, clinics, community centres - and home birth attendants must be also be the target for education about the harmful effects of prelacteal feed.

Introduction:

- Paragraph 2: Suggest rewording “Providing prelacteal ... is an established practice and other developing countries”.
- Paragraph 3: It is surprising that you refer to EBF till the recommended age. Be precise here. EBF is recommended for 6 months.
- Last sentence, “EBF among 3 month old children …”: Please replace “children” with “infants”.

 

 Discussion:

- Paragraph 2, line 6: “Our findings suggest that the families proving …” This is a typo. The word should be “providing”.
- Paragraph 3: Your concluding recommendation needs to be revised. Please see my earlier comments under Abstract and revise accordingly.
- Limitations: Please avoid using the term “economically backward region” - better to use “economically deprived region”.

I have read this submission. I believe that I have an appropriate level of expertise to confirm that it is of an acceptable scientific standard, however I have significant reservations, as outlined above.

# Response

**Published:** 2019-06-04

**Authors:** Aritra Das

Thank you Dr. Lartey for carefully reviewing the manuscript and for your valuable comments. As per your suggestion, we have made the following changes to the manuscript:

- In the first sentence of the ‘Methods’ section of the abstract, replaced ‘ *was’* with ‘ *were’*.
- In the ‘Conclusion’ section of the abstract, added the following sentence at the end – “ *Delivery conductors at the public and private facilities as well as the home birth attendants should also be made aware about the negative effects of prelacteal feed*”.
- In the first sentence of the second paragraph under ‘Introduction’, replaced ‘ *custom’* with ‘ *practice’* and ‘ *nation’* with ‘ *countries’*.
- In the second sentence of the third paragraph under ‘Introduction’, replaced ‘ *till recommended age’* with ‘ *sixth month’*.
- In the last sentence of the third paragraph under ‘Introduction’, replaced ‘ *children’* with ‘ *infants’*.
- In the second sentence of the second paragraph under ‘Discussion’, corrected the spelling error ‘ *providing’*.
- Added the following sentences at the end of the second paragraph of ‘Discussion’ section: “ *Another important intervention would be to intensify health education of would-be mothers, especially during pregnancy, and other family members such as their mothers-in-law about the harmful effects of prelacteal feeding for EBF and subsequently on infant health. Targeted education on the same topic for midwives/nurses working at the public and private hospitals and birth attendants conducting home deliveries may also prove useful in reducing the practice of prelacteal feeding*”.
- In the last sentence of the paragraph on ‘Limitations’ under ‘Discussion’ section, replaced ‘ *backward*’ with ‘ *deprived*’.

We fully agree with your comment on educating the mothers and the delivery conductors (nurses/midwives/home birth attendants) regarding the harmful effects of prelacteal feeding and we have added the recommendation in the manuscript. However, we have also retained the initial recommendation regarding identification and counseling of the mothers by Frontline workers (FLW). This recommendation is very context-specific as FLWs play a crucial role in providing health services in the state of Bihar, which has long been plagued by a weak health system. Not only do they have the widest reach (as almost 40% deliveries in Bihar take place either at home or at private facilities - which often remain untouched by the public health system), the FLWs can easily be influenced by the existing system in performing the desired task of counseling the mothers/family members on harmful effects of prelacteal feeding.

# Peer review: Reviewer response for version 2

**DOI:** 10.21956/gatesopenres.14009.r26896

**Published:** 2019-03-04

**Authors:** Manas Roy

**Affiliations:** 1 Directorate General of Health Services, Ministry of Health and Family Welfare, New Delhi, India

No further comments to make. The changes are satisfactory.

I have read this submission. I believe that I have an appropriate level of expertise to confirm that it is of an acceptable scientific standard.

# Peer review: Reviewer response for version 1

**DOI:** 10.21956/gatesopenres.13945.r26848

**Published:** 2019-01-11

**Authors:** Manas Roy

**Affiliations:** 1 Directorate General of Health Services, Ministry of Health and Family Welfare, New Delhi, India

**Comments**

- It is not clear why first three days after birth were excluded from working definition of EBF.
- 4 clients from 19 centres in 137 blocks – makes it 10412. How did the authors reach 10392 as sample size?
- In the definition of eligible household, it should be clarified that presence of child from ANY of the age groups was sufficient.
- Details about study participants are missing.
- ICDS is to be expanded. A line or two explaining how integration works between ASHA and ANM.
- Discussion does not include factors associated with EBF.
- The study involved children aged 3 months. How did recall bias for data on EBF for 6 months come to picture?

I have read this submission. I believe that I have an appropriate level of expertise to confirm that it is of an acceptable scientific standard, however I have significant reservations, as outlined above.

# Response

**Published:** 2019-01-11

**Authors:** Aritra Das

Dear Dr. Roy,

We express our sincere appreciation for your insightful comments. Please find below our responses to the queries posed by you:

It is not clear why first three days after birth were excluded from working definition of EBF.

*The operational definition of* *prelacteal* *feeding considered in this study is: "Providing something other than breast milk to an infant during the first three days of life". As the association of* *prelacteal* *feeding (exposure in this context) has been tested with exclusive breastfeeding (the 'outcome'), we excluded first three days of life from 'outcome' definition in order to maintain the temporal association between exposure and outcome. In other words, had we considered first 3 days of life for exclusive breastfeeding, then any provision of* *prelacteal* *feeding would have automatically resulted in non-exclusive breastfeeding and we could not have tested for the concerned hypothesis*.

4 clients from 19 centres in 137 blocks – makes it 10412. How did the authors reach 10392 as sample size.

*Thanks for pointing this out. We only considered the cases with complete information (for the parameters under consideration) for the analysis. For this reason, 20 cases had to be dropped as they had missing data for at least one or more variables.We will mention this in the *.  

In the definition of eligible household, it should be clarified that presence of child from ANY of the age groups was sufficient.

*Thank you for the suggestion. We will include the statement in the revised manuscript*.

Details about study participants are missing.

*We agree. We excluded the description in the current article as it has already been described in a previously published article (cited under 'Methods' - citation no. 12 - **Das A, Chatterjee R, Karthick M, et al.: The Influence of Seasonality and Community-Based Health Worker Provided Counselling on Exclusive Breastfeeding - Findings from a Cross-Sectional Survey in India. PLoS One. 2016; 11(8).*** *) .* *As per suggestion, we will add a sentence in the results section stating that the "characteristics of the study population* *has* *been described in a previous article"*.

ICDS is to be expanded. A line or two explaining how integration works between ASHA and ANM.

*Thank you for the suggestion. We will include the full form of ICD in the revision. We will also add the following sentence to describe the relationship between ASHA and ANM - "ASHA workers deliver various MNCH and immunization service at the village level. The Auxilliary Nurse Midwives or ANMs are the key health functionary at the Health Sub-centre (HSC) level (consisting of several villages) with a broad set of responsibilities, including the support, local supervision and capacity building of the ASHA and AWW working in respective HSC catchment areas"*.

The study involved children aged 3 months. How did recall bias for data on EBF for 6 months come to picture?

*Although the study involved interviews with mothers of infants aged 0-2 completed months, there is unlikely to be any differential recall between mothers of neonates and those with older (up to 3 months old) infants. This is due to the fact that the data on EBF is based on 24 hours recall. Also, prelacteal feeding in rural Bihar is often based on family/social customs and the mothers are unlikely to forget the feeding given to their child during first few days of life. However, as per suggestion, we will include this caveat under Discussion section in the revised manuscript.*
